# Impact of combined balance and strength exercise program on lower limb energy flow in individuals with knee osteoarthritis

**DOI:** 10.3389/fspor.2025.1661125

**Published:** 2025-10-09

**Authors:** Ponthep Tangkanjanavelukul, Nattapat Khumtong, Khemchat Chaemklan, Dipak Kumar Agrawal, Pornthep Rachnavy

**Affiliations:** ^1^ Institute of Medicine, Suranaree University of Technology, Nakhon Ratchasima, Thailand; ^2^ Department of Interdisciplinary Science and Internationalization, Institute of Science, Suranaree University of Technology, Nakhon Ratchasima, Thailand; ^3^ School of Telecommunication Engineering, Faculty of Engineering, Suranaree University of Technology, Nakhon Ratchasima, Thailand; ^4^ School of Sports Science, Institute of Science, Suranaree University of Technology, Nakhon Ratchasima, Thailand

**Keywords:** biomechanics, exercise therapy, joint stability, lower extremity, knee osteoarthritis

## Abstract

**Introduction:**

Knee osteoarthritis disrupts biomechanical energy flow, resulting in joint instability, impaired movement, and pain. These issues impact daily activities and increase the risk of falls. Effective interventions are essential.

**Objective:**

This study examined the effects of a six-month balance and strength training program on lower limb biomechanics and self-reported outcomes in individuals with mild to moderate knee osteoarthritis.

**Methods:**

Twenty-three participants (mean age: 62.4 years; 69.57% female) completed a structured balance and strength exercise program three times per week. Gait analysis was used to assess lower limb energy flow, while the Western Ontario and McMaster Universities Osteoarthritis Index (WOMAC) measured symptoms before and after the intervention.

**Results:**

Significant improvements were observed in overall WOMAC scores (*p* = 0.009), including subscales for pain (*p* = 0.022), stiffness (*p* = 0.005), and physical function (*p* = 0.013). Energy flow analysis revealed increased energy inflow and outflow at the hip (*p* < 0.001), reduced energy absorption at the knee (*p* < 0.001), and enhanced energy outflow at the ankle (*p* < 0.001), suggesting improved gait dynamics.

**Discussion:**

The combined balance and strength exercise program effectively enhanced lower limb biomechanics and reduced knee osteoarthritis symptoms. Energy flow analysis may support personalized rehabilitation approaches and help identify individuals at elevated fall risk.

**Conclusion:**

This exercise program improved lower limb biomechanics, reduced pain and stiffness, enhanced energy flow, and may optimize rehabilitation and fall prevention in individuals with knee osteoarthritis.

## 1 Introduction

Millions of people around the world are affected by Knee Osteo-Arthritis (KOA), which is one of the leading causes of pain and disability (1). The condition disrupts mechanical energy transfer among the hip, knee, and ankle joints, critical components of efficient movement and energy conservation. This disruption manifests as altered movement mechanics, increased joint loading, and pain (2–4). KOA impairs the knee’s ability to absorb and generate energy, leading to compensatory stress on the hip and ankle joints, inefficient joint coordination, and reduced gait efficiency (5). Analyzing energy transfer patterns provides a basis for developing non-surgical treatments, including combined balance and strength exercise programs that aim to improve joint function and reduce pain and instability.

Exercise-based rehabilitation is a cornerstone of KOA management, offering a non-invasive approach to relieve pain and enhance function (6, 7). Programs commonly incorporate strength training and balance exercises to enhance muscle force production and neuromuscular control (8). However, the specific effects of these interventions on energy flow dynamics, including how energy is absorbed, generated, and transferred across joints, remain poorly understood, particularly in cases of mild to moderate KOA. Although combined balance and strength programs are well-established for improving balance and strength in older adults, their influence on energy flow patterns in KOA patients has not been systematically investigated (9). Addressing this gap is important, as understanding how these programs influence energy dynamics could provide insights into their mechanisms of action and help optimize their clinical application for KOA.

The use of advanced biomechanical technologies, such as three-dimensional motion capture and force platforms, is valuable for analyzing energy flow parameters (inflow, outflow, absorption, and generation) during dynamic tasks like gait in individuals with KOA (5, 10, 11). Unlike traditional assessments focused solely on kinematics or kinetics, these tools enable a more nuanced understanding of how energy is transferred and dissipated across joints, offering a comprehensive evaluation of movement efficiency. By integrating advanced statistical techniques such as Principal Component Analysis and Hierarchical Cluster Analysis, these tools enable the identification of subtle yet clinically meaningful changes in energy flow patterns following interventions.

This study investigates the impact of a six-month combined balance and strength exercise program on hip, knee, and ankle energy flow dynamics during gait in individuals with mild to moderate KOA. By assessing energy inflow, outflow, absorption, and generation before and after the intervention, we aim to identify joint-specific factors contributing to improved movement efficiency and stability. For example, reduced energy absorption at the knee may indicate better load distribution, while increased energy generation at the ankle may reflect enhanced push-off strength. These biomechanical adaptations are expected to correlate with patient-reported pain, stiffness, and function improvements, as measured by the Western Ontario and McMaster Universities Osteoarthritis Index (WOMAC). By establishing a clear relationship between energy flow adaptations and clinical outcomes, this study aims to generate new insights that support the design of more effective non-surgical interventions for KOA.

## 2 Methods

### 2.1 Study design and participants

#### 2.1.1 Study design

The researchers employed a longitudinal, single-group, pre-post intervention study design to investigate the impacts of a six-month combined balance and strength exercise program on individuals with mild to moderate knee osteoarthritis. Participants underwent assessments before and after the intervention, which included gait analysis with motion capture, force plate measurements of lower limb energy flow, and completion of the Western Ontario and McMaster Universities Osteoarthritis Index (WOMAC) questionnaire.

#### 2.1.2 Participants

Twenty-three individuals diagnosed with mild to moderate Knee Osteo-Arthritis (KOA) were recruited from outpatient physical therapy clinics and hospital-based rehabilitation facilities in Nakhon Ratchasima, Thailand. Participant eligibility was based on clinical and radiographic criteria established by the American College of Rheumatology (ACR). Inclusion criteria required participants to be aged 60–85 years, capable of walking independently for at least 10 m without assistive devices, and free from significant lower extremity surgeries (e.g., total knee arthroplasty) or neurological conditions affecting gait.

Exclusion criteria included the presence of severe comorbidities affecting gait (e.g., stroke, uncontrolled cardiovascular or pulmonary disease, or diabetes), inability to comply with the exercise protocol, or receipt of gait-affecting treatments (e.g., intra-articular injections, surgery, or physical therapy) within the previous three months. Final diagnoses were confirmed via clinical assessments and medical record reviews by qualified physicians.

#### 2.1.3 Sample size and power analysis

To determine an appropriate sample size, we referred to (12), who reported a large effect size (*d* = 1.92) on WOMAC outcomes following a 12-week combined balance and strength training program. Using G*Power 3.1, an a priori power analysis (sample size calculation performed prior to data collection) based on a two-tailed *t*-test (α = 0.05, power = 0.80) indicated a minimum sample size of five participants. To ensure statistical power and account for potential attrition or biological variability, we recruited 23 participants substantially exceeding the minimum threshold to reduce the risk of Type II error (13).

#### 2.1.4 Ethical approval

The research protocol was reviewed and approved by the institutional ethics board of Suranaree University of Technology (EC-62-0094). All participants provided written informed consent prior to their enrollment.

### 2.2 Intervention protocol

#### 2.2.1 Combined balance and strength exercise program

Participants engaged in a six-month, home-based intervention following a combined balance and strength exercise protocol tailored for individuals with Knee Osteo-Arthritis (KOA). This evidence-based program, grounded in neuromuscular control and postural stability principles, was designed to enhance quadriceps strength, neuromuscular coordination, and joint stability. Participants completed three sessions per week, each lasting 30 to 60 min.

Each session began with a 5–10 min warm-up involving light aerobic activities such as brisk walking and dynamic range-of-motion movements, including shoulder rolls, knee extensions, and ankle pumps. The core 20–50 min training phase included multi-planar lower extremity strengthening exercises, specifically front knee lifts targeting the quadriceps, side hip lifts focusing on the gluteus medius, back knee lifts with slight flexion engaging the hamstrings and glutes, calf and toe raises for the gastrocnemius and tibialis anterior, controlled knee bends for eccentric quadriceps strengthening, and functional sit-to-stand transitions to improve core and quadriceps control.

Balance components of the program included heel-to-toe walking (tandem gait), one-leg standing for static balance, and backward walking to challenge proprioception. Exercise intensity progressed over time through increases in sets and repetitions, as shown in Table 1. All adaptations-maintained fidelity to exercise training principles while accommodating KOA-specific biomechanical requirements.

**Table 1:** Exercise intensity progression for combined balance and strength program.

Exercise	Week 1–4	Week 4–8	Week 8–12	Week 12–16	Week 16–24
Front knee strengthening (sets × reps)	(1 × 10)	(2 × 10)	(2 × 10)	(3 × 12)	(3 × 15)
Side hip strengthening (sets × reps)	(1 × 10)	(2 × 10)	(2 × 10)	(3 × 12)	(3 × 15)
Back knee strengthening (sets × reps)	(1 × 10)	(2 × 10)	(2 × 10)	(3 × 12)	(3 × 15)
Calf raises (sets × reps)	(1 × 10)	(2 × 10)	(2 × 10)	(3 × 12)	(3 × 15)
Toe raises (sets × reps)	(1 × 10)	(2 × 10)	(2 × 10)	(3 × 12)	(3 × 15)
Knee bends (sets × reps)	(1 × 10)	(2 × 10)	(2 × 10)	(3 × 12)	(3 × 15)
Sit-to-stand (sets × reps)	(1 × 10)	(2 × 10)	(2 × 15)	(3 × 12)	(3 × 15)
Heel-toe walking (sets × steps)	(1 × 10)	(2 × 10)	(2 × 15)	(3 × 12)	(3 × 15)
One-leg stand (sets × seconds)	(1 × 10)	(2 × 10)	(2 × 20)	(3 × 15)	(3 × 20)
Backward walking (sets × steps)	(1 × 10)	(2 × 10)	(2 × 15)	(3 × 12)	(3 × 15)

To further tailor the program, all exercises emphasized low-impact movement to minimize joint stress, with special attention to strengthening the quadriceps and hamstrings for improved knee stability (7). Balance exercises were adjusted to participant capacity, such as increasing the base of support to reduce fall risk during unilateral stance tasks. To ensure adherence, participants received weekly phone calls from the research team to offer motivation, address concerns, troubleshoot difficulties, and review proper exercise techniques. Additionally, each participant maintained an exercise log to track session completion, recording exercise type, number of sets and repetitions, and perceived exertion levels.

**Figure 1:**
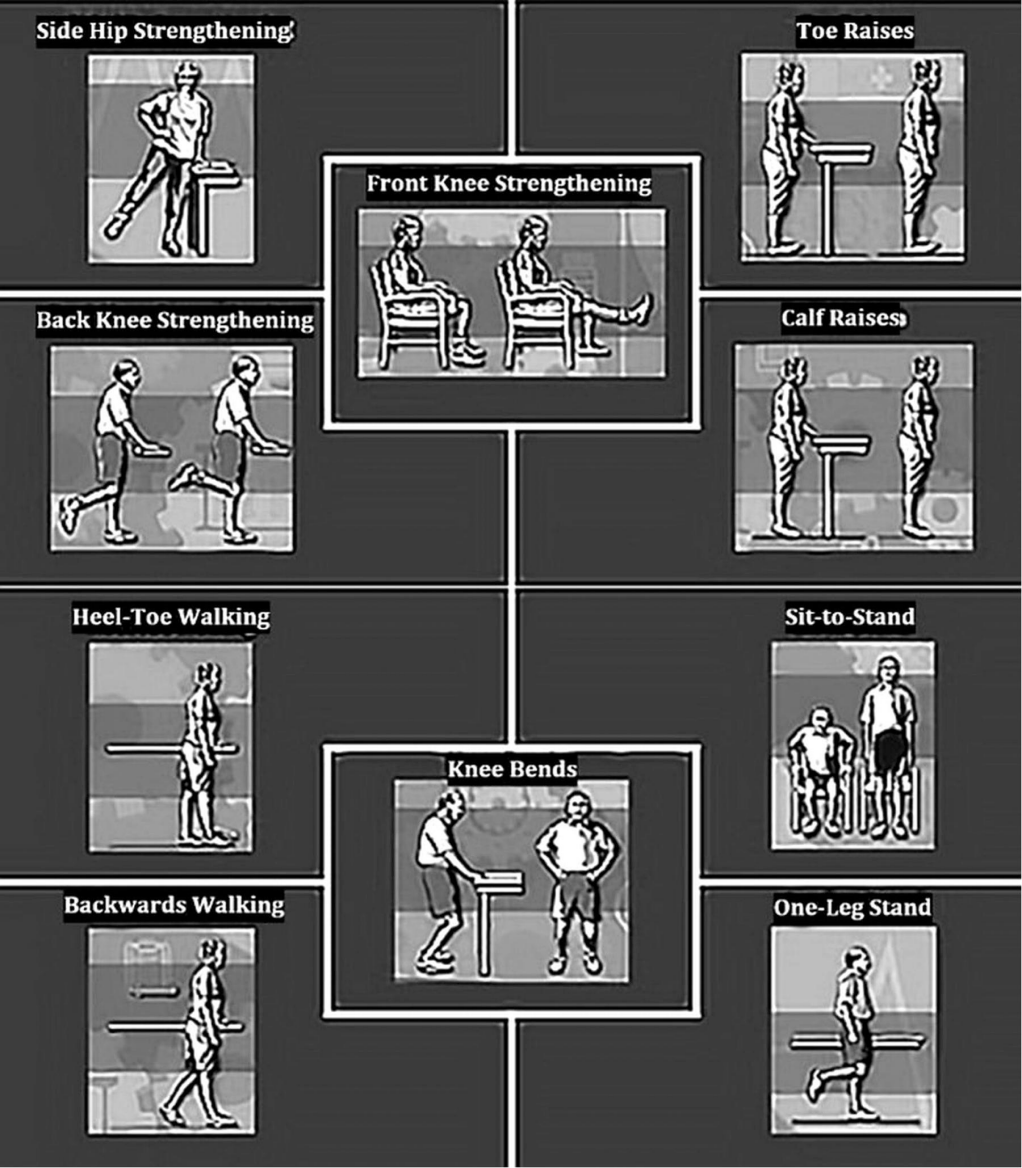
The combined balance and strength exercise intervention.

This intervention, illustrated in Figure 1, reflects a structured fall-prevention model consistent with evidence-based programs such as the Otago Exercise Program, which emphasizes strength and balance training as a proven strategy for reducing fall risk in older adults (14).

### 2.3 Data collection and processing

#### 2.3.1 Gait analysis procedure

Prior to data collection, participants were prepared by wearing comfortable clothing and athletic shoes. Reflective markers were affixed to anatomical landmarks using a standardized marker set protocol. Participants completed a static calibration trial, followed by at least three walking trials at a self-selected pace to reflect a natural gait pattern.

The gait cycle was defined as the interval from heel strike to subsequent heel strike of the same foot, with initial contact determined by a force plate threshold of 20 N. This threshold was established based on pilot testing and aligns with previous literature. Gait parameters were analyzed in accordance with established biomechanical guidelines. The relevance of gait analysis to exercise-based rehabilitation, a cornerstone of KOA management, has been well-documented (15). While this study focused on KOA, it is acknowledged that coexisting conditions such as hip osteoarthritis may also influence gait patterns (16).

##### 2.3.1.1 Motion capture system

Three-dimensional kinematic data were collected using a six-camera Qualisys Miqus motion capture system (Qualisys AB, Sweden), operating at a resolution of 2 megapixels and a frame rate of 200 Hz. The cameras were arranged around a 5 m × 2 m capture volume to ensure comprehensive tracking of participant movement. Data acquisition and preliminary processing were performed using Qualisys Track Manager software (version 2021.1). Both static and dynamic wand calibrations were completed before each session to ensure system accuracy.

##### 2.3.1.2 Force plate specifications

Kinetic data were obtained via two Kistler 9286BA force plates (Kistler Group, Switzerland), measuring 600 mm × 400 mm. These force plates were embedded side-by-side within the walkway to capture bilateral ground reaction forces during gait. Data from the force plates were sampled at 200 Hz to match the motion capture system.

##### 2.3.1.3 Data synchronization

Kinematic and kinetic data streams were synchronized using a hardware-based synchronization unit that generated TTL (transistor-transistor logic) trigger signals to align both systems temporally. This approach ensured precise frame-to-frame synchronization, consistent with best practices in biomechanical gait analysis (17).

#### 2.3.2 Marker placement

Thirty-five retro-reflective markers (14 mm in diameter) were strategically placed on specific anatomical landmarks according to a standardized skin marker set protocol. To ensure consistency and minimize inter-rater variability, a single-trained researcher affixed all markers using double-sided adhesive tape (18). The placement of the markers adhered to the following anatomical configuration: **Head:** Markers were positioned at the center of the frontal bone and bilaterally on the parietal bones.

**Upper body:** Markers were placed on the right and left acromion processes, the sternum, cervical vertebra C2, and thoracic vertebra T12.

**Pelvis:** Markers were affixed to the right and left pubic bones and at the center of the sacrum.

**Upper limbs:** Markers were positioned on coronoid fossae, medial and lateral epicondyles, lunate bones, ulnae, and radii bilaterally.

**Lower limbs:** Markers were attached to the lateral collateral ligaments, centers of the patellae, tibial heads, third and fifth metatarsals, calcanei, and tali.

**Figure 2:**
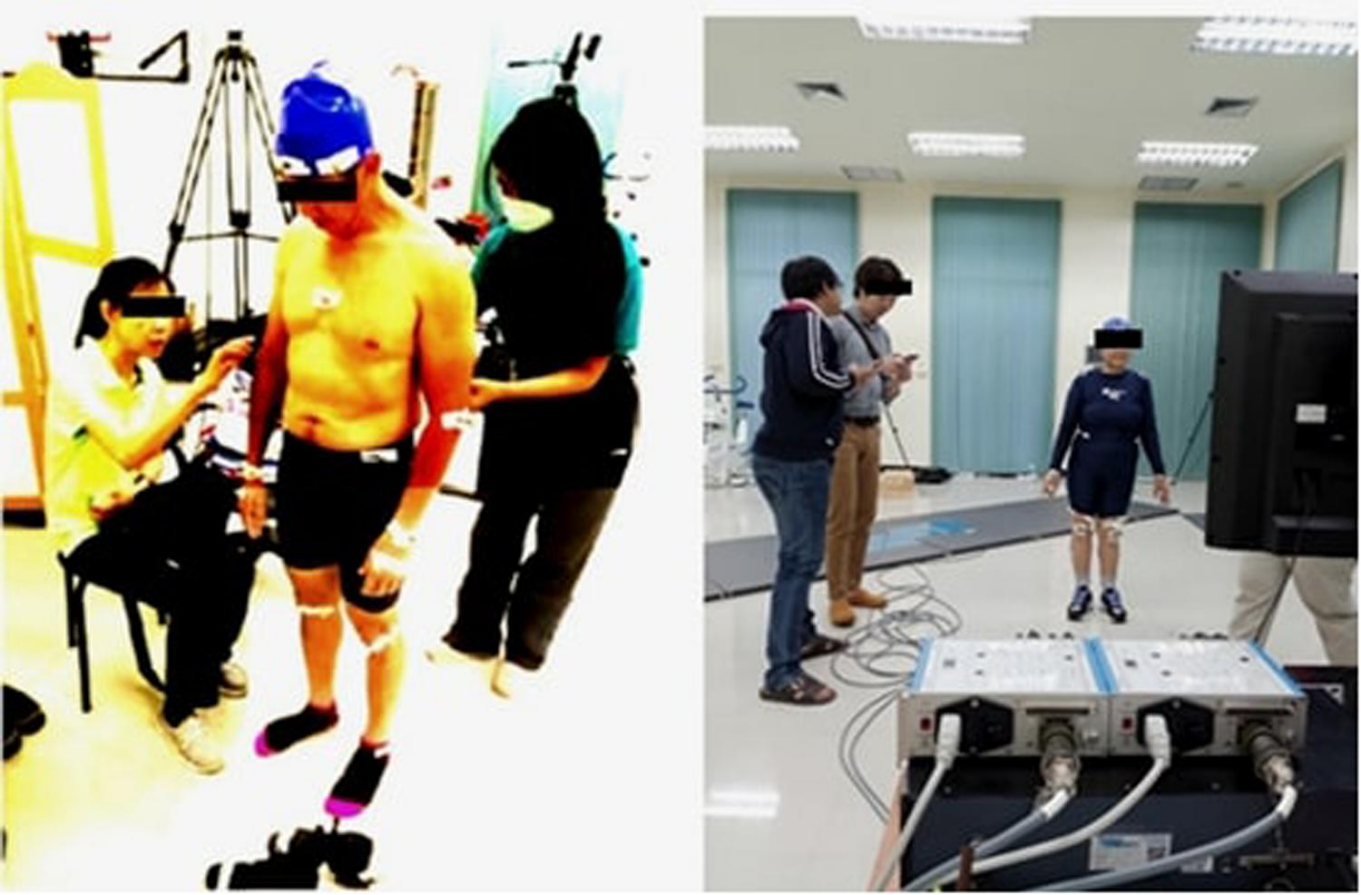
Marker placements on anatomical reference points used for motion capture.

This systematic positioning permitted the calculation of joint angles across the sagittal, frontal, and transverse planes. Careful palpation of anatomical landmarks was conducted to limit soft tissue artifacts. Marker placement adhered to established biomechanical protocols, thus facilitating reliable three-dimensional motion analysis across all body segments. A static calibration trial was executed before conducting dynamic trials to define subject-specific anatomical coordinate systems. The specific marker locations are illustrated in Figure 2.

#### 2.3.3 Biomechanical modeling

A 14-segment model with 26 degrees of freedom was employed to analyze joint-level biomechanics using Visual3D software (C-Motion, Germantown, USA, v2021.11.3). By leveraging the Inverse Dynamics and Segmental Power Analysis modules, we computed joint forces (N), segmental velocities (m/s), joint moments (N m), and angular velocities (rad/s). The outputs generated were crucial in characterizing energy flow—specifically, energy inflow, outflow, absorption, and generation—at the hip, knee, and ankle joints.

**Figure 3:**
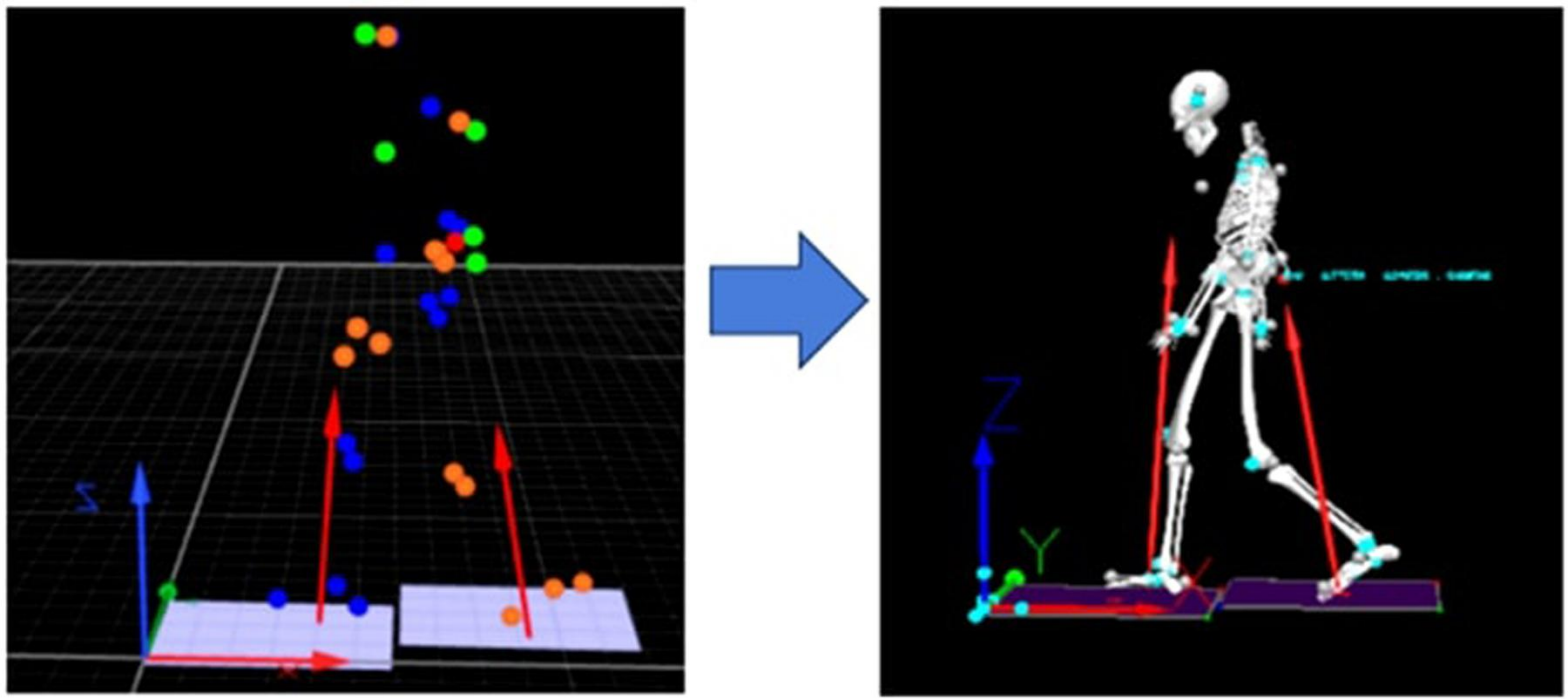
Three-dimensional marker trajectories captured by the Qualisys motion analysis system during gait.

Marker trajectories were recorded using a six-camera Qualisys Miqus motion analysis system (Qualisys AB, Sweden) operating at 200 Hz. This high-speed optical system enabled the capture of precise three-dimensional coordinates for each marker within a calibrated measurement volume. The synchronized marker data were subsequently imported into Visual3D for comprehensive biomechanical modeling and analysis. The spatial arrangement of the markers, as tracked by the Qualisys system, is visualized in Figure 3.

#### 2.3.4 Energy flow computations

Joint angles and segmental movements were calculated from the marker trajectories. Joint forces (F), linear joint velocity (V), joint moments (T), and segment angular velocity (ω) were computed using Visual3D software. These parameters were used to calculate joint power, segmental torque power, and overall segmental power to quantify energy flow at the ankle, knee, and hip joints.

The following equations were used:

• **Joint force power (JFP):**
  (1) $$\text{JFP}=F_{j}\cdot V_{j}$$
• **Segmental torque power (STP):**
  (2) $$\text{STP}=T_{j}\cdot \omega_{s}$$
• **Segmental power (SP):**
  (3) $$\text{SP}={\text{JFP}}_{d}+{\text{JFP}}_{p}+{\text{STP}}_{d}+{\text{STP}}_{p}$$

where $F_{j}$ is the joint reaction force, $V_{j}$ is the linear joint velocity, $T_{j}$ is the joint moment, $\omega_{s}$ is the segment angular velocity, and subscripts d and p denote the distal and proximal ends of the segment, respectively.

Energy transfer between adjacent segments (ankle-to-knee and knee-to-hip) was calculated as the rate of energy flow into or out of a body segment. This was computed using segmental power (SP, Equation 3), derived from the combination of joint force power (JFP, Equation 1) and segmental torque power (STP, Equation 2), with the subscripts d and p representing the distal and proximal ends of the segment, respectively.

**Figure 4:**
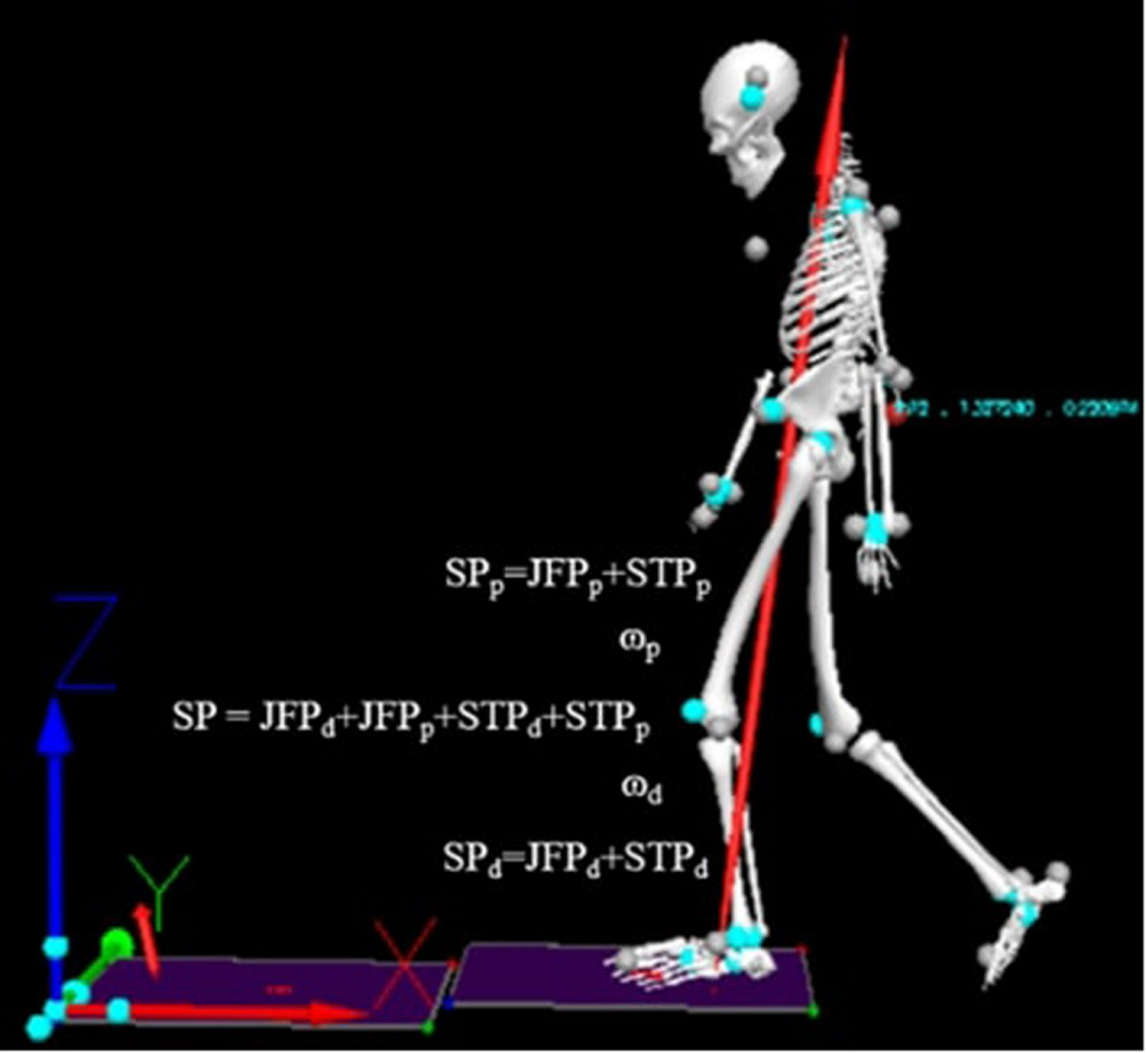
Calculation workflow for joint force power (JFP), segmental power due to torque (STP), and overall segmental power (SP) used to quantify energy flow at the ankle, knee, and hip joints.

As illustrated in Figure 4, the biomechanical analysis in this study was conducted using Visual3D software to process motion capture data acquired from the Qualisys system. The recorded marker trajectories enabled the calculation of essential biomechanical parameters, including joint forces (N), joint moments (N.m), and angular velocities (rad/s) of body segments. These parameters were then used to compute joint power (JFP), torque-based segmental power (STP), and total segmental power (SP). Collectively, these metrics provided detailed insights into the mechanical energy flow and joint dynamics during gait, supporting the interpretation of functional changes resulting from the intervention.

### 2.4 Outcome measures and statistical analysis

#### 2.4.1 WOMAC assessment

Functional outcomes were assessed using the Western Ontario and McMaster Universities Osteoarthritis Index (WOMAC), version 3.1. This validated instrument evaluates three subscales: pain (0–20), stiffness (0–8), and physical function (0–68), yielding a total score range of 0–96, with higher scores indicating greater levels of impairment (19–22).

The WOMAC 3.1 has demonstrated strong psychometric properties in populations with knee osteoarthritis. Prior studies involving individuals with mild to moderate KOA have consistently reported Cronbach’s alpha values exceeding 0.90 across all subscales, indicating excellent internal consistency and reliability (23).

Participants completed the WOMAC questionnaire at baseline (pre-intervention) and again at the six-month follow-up (post-intervention). The questionnaire was primarily self-administered; however, trained research staff were available to assist participants when necessary to ensure the accuracy and consistency of responses.

#### 2.4.2 Statistical analysis

All data were analyzed using SPSS Software (Version 27, IBM, USA). Descriptive statistics were computed as (means ± standard deviations) for all variables. The Shapiro–Wilk test assessed the normality of data distributions. Paired *t*-tests compared pre- and post-intervention values for normally distributed variables. Effect sizes were interpreted using Cohen’s *d*, with thresholds of 0.2–0.49 indicating a small effect, 0.5–0.79 a medium effect, and ≥ 0.8 a large effect.

Principal Component Analysis (PCA) with varimax rotation was applied to reduce the dimensionality of mechanical energy flow and gait parameters. Components were retained according to the Kaiser criterion (eigenvalues > 1) (24, 25). PCA was chosen for its ability to identify underlying patterns in complex biomechanical data, facilitating the interpretation of energy flow dynamics across joints.

A hierarchical clustering (Ward’s linkage, squared Euclidean distance) approach was employed to categorize participants into groups based on characteristics of mechanical energy flow. Ward’s linkage was selected to minimize within-cluster variance, while squared Euclidean distance was chosen as it is suitable for continuous biomechanical data and assumes clusters of similar sizes and shapes. This method was selected for its ability to group participants with similar biomechanical profiles, providing insights into potential subgroups that may respond differently to the intervention. All statistical tests were conducted at a significance level of p<0.05.

## 3 Results

This study investigated changes in mechanical energy flow at the hip, knee, and ankle joints before and after a six-month combined balance and strength exercise program using Principal Component Analysis (PCA) and Hierarchical Clustering to explore trends in energy dynamics. Functional outcomes were assessed using the Western Ontario and McMaster Universities Osteoarthritis Index (WOMAC), which evaluated changes in pain, stiffness, and physical function in individuals with knee osteoarthritis.

### 3.1 Participant demographics

The study sample consisted of 23 individuals, with 7 male and 16 female participants. The demographic data for the participants is summarized in Table 2. The mean age was 62.38 ± 9.1 years, ranging from 60 to 85 years. Participants had a mean body weight of 63.87 ± 8.65 kg and a mean height of 157.23 ± 5.36 cm, resulting in an average body mass index (BMI) of 25.79 ± 2.88 ${\mathrm{kg}/m}^{2}$. Observed BMI values ranged from 21.1 to 31.2.

**Table 2:** Demographic profile of study participants.

Characteristic	Mean	SD	Range
Age (years)	62.38	9.10	60–85
Weight (kg)	63.87	8.65	50–85
Height (cm)	157.23	5.36	145–165
BMI (kg/m^2^)	25.79	2.88	21.1–31.2

### 3.2 Paired samples test results

To evaluate the effectiveness of the intervention, paired-samples *t*-tests were performed to compare pre- and post-intervention measurements across key outcome parameters. Table 3 summarizes the results, including pre-test and post-test means, mean differences, standard deviations (SD), *t*-values, degrees of freedom (df), and *p*-values for each parameter.

**Table 3:** Paired-samples *t*-test results comparing pre- and post-intervention outcomes (*n* = 23).

Paired variables	Pre-test	Post-test	Mean diff	SD	*t*	df	*p*-value	Cohen’s *d*
WOMAC score overall	75.00	48.52	26.48	44.18	2.87	22	.009	0.60
WOMAC pain	14.43	9.22	5.22	10.11	2.47	22	.022	0.52
WOMAC stiffness	6.43	3.52	2.91	4.47	3.13	22	.005	0.65
WOMAC function	54.13	35.61	18.52	32.96	2.70	22	.013	0.56
Ankle energy inflow (W/kg)	0.9763	1.1132	−0.137	0.155	−4.244	22	<0.001	0.88
Ankle energy outflow (W/kg)	1.8254	1.5304	0.295	0.059	24.01	22	<0.001	4.99
Ankle energy absorption (W/kg)	0.6530	0.6020	0.051	0.060	4.047	22	0.001	0.85
Ankle energy generation (W/kg)	1.8990	1.6119	0.287	0.172	8.018	22	<0.001	1.67
Knee energy inflow (W/kg)	0.6763	1.0132	−0.337	0.155	−10.443	22	<0.001	2.18
Knee energy outflow (W/kg)	0.6090	0.9639	−0.355	0.143	−11.94	22	<0.001	2.48
Knee energy absorption (W/kg)	0.5894	0.8674	−0.278	0.039	−34.282	22	<0.001	7.13
Knee energy generation (W/kg)	0.5847	0.9878	−0.403	0.098	−19.819	22	<0.001	4.11
Hip energy inflow (W/kg)	0.6304	0.4905	0.140	0.118	5.705	22	<0.001	1.19
Hip energy outflow (W/kg)	1.2148	1.1481	0.067	0.077	4.162	22	<0.001	0.87
Hip energy absorption (W/kg)	0.5703	0.4119	0.158	0.148	5.141	22	<0.001	1.07
Hip energy generation (W/kg)	1.2518	1.1380	0.114	0.218	2.497	22	0.02	0.52

Significant reductions in WOMAC scores indicated improvements in joint function, pain, stiffness, and mobility following the intervention. The total WOMAC score decreased from 75.00 to 48.52 (mean difference = 26.48, *p* = 0.009, Cohen’s *d* = 0.60, representing a moderate to large effect), with notable reductions in pain (mean difference = 5.22, *p* = 0.022, Cohen’s *d* = 0.52, indicating a moderate effect), stiffness (mean difference = 2.91, *p* = 0.005, Cohen’s *d* = 0.65, representing moderate to large effect), and physical function limitations (mean difference = 18.52, *p* = 0.013, Cohen’s *d* = 0.56, indicating a moderate effect). These results support the effectiveness of the Otago Exercise Program (OEP) in alleviating Knee Osteo-Arthritis (KOA) symptoms.

Paired *t*-tests revealed significant changes in mechanical energy flow at the ankle, knee, and hip joints after the six-month combined balance and strength exercise program. The ankle showed increased energy inflow (mean difference = −0.137 W/kg, p<0.001, Cohen’s *d* = 0.88, representing a large effect) and decreased energy outflow (mean difference = 0.295 W/kg, p<0.001, Cohen’s *d* = 4.99, indicating an exceptionally large effect), absorption (mean difference = 0.051 W/kg, *p* = 0.001, Cohen’s *d* = 0.85, representing a large effect), and generation (mean difference = 0.287 W/kg, p<0.001, Cohen’s *d* = 1.67, indicating a very large effect).

At the knee, energy inflow (mean difference = −0.337 W/kg, p<0.001, Cohen’s *d* = 2.18, indicating a very large effect), outflow (mean difference = −0.355 W/kg, p<0.001, Cohen’s *d* = 2.48, representing a very large effect), absorption (mean difference = −0.278 W/kg, p<0.001, Cohen’s *d* = 7.13, indicating an exceptionally large effect), and generation (mean difference = −0.403 W/kg, p<0.001, Cohen’s *d* = 4.11, representing a very large effect) all decreased significantly.

The hip exhibited a significant decrease in energy inflow (mean difference = 0.14 W/kg, p<0.001, Cohen’s *d* = 1.19, indicating a large effect) and absorption (mean difference = 0.158 W/kg, p<0.001, Cohen’s *d* = 1.07, representing a large effect), with a smaller but statistically significant reduction in energy generation (mean difference = 0.114 W/kg, *p* = 0.02, Cohen’s *d* = 0.52, indicating a moderate effect).

### 3.3 Principal component analysis (PCA)

To reduce dataset dimensionality and identify underlying patterns in mechanical energy variables, Principal Component Analysis (PCA) was performed. The analysis included 24 input variables related to energy inflow, outflow, absorption, and generation across the hip, knee, and ankle joints. Four principal components were extracted based on the Kaiser criterion (eigenvalues > 1.0).

These four components accounted for 90.71% of the total variance, indicating a strong cumulative explanatory power and a well-structured dimensionality reduction. The component loadings and explained variance for each factor are summarized in Table 4.

i. **Principal component 1 (PC1): hip-ankle synergy in energy transfer**
  PC1 explained 37.82% of the variance and captured energy inflow and outflow variables at the hip and ankle joints. The specific component loadings are detailed in Table 4.
ii. **Principal component 2 (PC2): knee’s role in shock absorption**
  PC2 accounted for 32.33% of the variance and correlated strongly with knee energy absorption, as shown in Table 4.
iii. **Principal component 3 (PC3): differential energy generation across joints**
  PC3 explained 11.83% of the variance and reflected variations in energy generation across the hip, knee, and ankle joints. Details of these variations are presented in Table 4.
iv. **Principal component 4 (PC4): individual variations in energy absorption**
  PC4 contributed 8.72% of the variance and captured unique energy absorption patterns. Further details can be found in Table 4.

**Table 4:** Total variance explained by the four principal components derived from PCA (*n* = 24 variables).

Component	Initial eigenvalues	% of variance	Cumulative %	Rotation sums of squared loadings	% of variance	Cumulative %
1	17.336	72.23%	72.23%	9.077	37.82%	37.82%
2	1.720	7.17%	79.40%	7.760	32.33%	70.15%
3	1.454	6.06%	85.46%	2.839	11.83%	81.98%
4	1.260	5.25%	90.71%	2.094	8.72%	90.71%

### 3.4 Rotated component matrix analysis

Table 5 presents the result of the rotated component matrix, showing energy flow variables across the ankle, knee, and hip joints loading onto four components, representing distinct patterns of energy inflow, outflow, generation, and absorption during pre- and post-test conditions.

i. **Component 1 (C1): baseline energy dynamics**
  Component 1 (C1) showed high loadings for pre-intervention energy inflow, outflow, and absorption variables across the ankle, knee, and hip joints (e.g., ankle inflow pre-test: 0.907; hip inflow pre-test: 0.862; hip absorption pre-test: 0.869). Full component loadings are presented in Table 5.
ii. **Component 2 (C2): post-intervention energy transfer**
  Component 2 (C2) was characterized by high loadings for post-intervention energy inflow and outflow variables (e.g., ankle inflow post-test: 0.851; hip inflow post-test: 0.841). See Table 5 for all loadings.
iii. **Component 3 (C3): pre-test energy generation**
  Component 3 (C3) reflected energy generation variables at the ankle and knee during baseline conditions, with strong loadings for the ankle generation pre-test and knee outflow pre-test (0.897). Details are in Table 5.
iv. **Component 4 (C4): absorption and redistribution patterns**
  Component 4 (C4) was associated with energy dissipation patterns, showing negative loadings for knee absorption pre-test (−0.898) and hip generation post-test (−0.601). Refer to Table 5 for comprehensive loadings.

**Table 5:** Rotated component matrix from principal component analysis of energy flow and gait variables.

Variables	Component 1	Component 2	Component 3	Component 4
Ankle energy inflow (pre-test)	0.907	0.386	0.094	0.109
Ankle energy inflow (post-test)	0.428	0.851	0.255	0.109
Ankle energy outflow (pre-test)	0.869	0.399	0.185	0.163
Ankle energy outflow (post-test)	0.698	0.659	0.194	0.175
Ankle energy absorption (pre-test)	0.612	0.336	0.240	0.011
Ankle energy absorption (post-test)	0.671	0.443	0.232	−0.067
Ankle energy generation (pre-test)	0.296	0.306	0.897	0.079
Ankle energy generation (post-test)	0.415	0.863	0.205	0.119
Knee energy inflow (pre-test)	0.907	0.386	0.094	0.109
Knee energy inflow (post-test)	0.428	0.851	0.255	0.109
Knee energy outflow (pre-test)	0.296	0.306	0.897	0.079
Knee energy outflow (post-test)	0.536	0.654	0.187	0.247
Knee energy absorption (pre-test)	0.085	−0.028	−0.129	−0.898
Knee energy absorption (post-test)	−0.264	−0.079	0.104	−0.741
Knee energy generation (pre-test)	0.749	0.412	0.469	0.167
Knee energy generation (post-test)	0.495	0.812	0.208	0.186
Hip energy inflow (pre-test)	0.862	0.404	0.251	0.135
Hip energy inflow (post-test)	0.461	0.841	0.237	0.145
Hip energy outflow (pre-test)	0.787	0.411	0.445	0.056
Hip energy outflow (post-test)	0.486	0.816	0.240	0.125
Hip energy absorption (pre-test)	0.869	0.392	0.127	0.187
Hip energy absorption (post-test)	0.415	0.863	0.205	0.119
Hip energy generation (pre-test)	0.849	0.415	0.267	0.105
Hip energy generation (post-test)	−0.134	−0.375	−0.166	−0.601

### 3.5 Component transformation matrix

The component transformation matrix (Table 6) provides valuable insights into the energy dynamics across the lower extremities, revealing distinct patterns of energy flow and dissipation during movement. Here is a short description of the results from Table 6:

i. **PC1**
  PC1 showed strong positive loadings for ankle and hip energy inflow/outflow variables (e.g., ankle outflow pre-test: 0.937; hip inflow pre-test: 0.951).
ii. **PC2**
  PC2 exhibited strong associations with knee energy absorption variables (e.g., pre-test: 0.813; post-test: 0.534).
iii. **PC3**
  PC3 showed moderate loadings from energy inflow and absorption variables, such as knee absorption post-test (0.49).
iv. **PC4**
  PC4 captured localized energy dynamics, with minor loadings from knee absorption and other joint-specific variables.

**Table 6:** Component transformation matrix of energy flow variables.

Variable	PC1	PC2	PC3	PC4
Ankle energy inflow (pre-test)	0.917	0.215	−0.323	0.019
Ankle energy inflow (post-test)	0.931	−0.124	0.245	0.208
Ankle energy outflow (pre-test)	0.937	0.147	−0.270	−0.053
Ankle energy outflow (post-test)	0.988	−0.008	−0.055	0.102
Knee energy absorption (pre-test)	−0.151	0.813	0.262	0.279
Knee energy absorption (post-test)	−0.326	0.534	0.490	0.071
Hip energy inflow (pre-test)	0.951	0.167	−0.216	−0.096
Hip energy inflow (post-test)	0.948	−0.136	0.196	0.205

### 3.6 Hierarchical clustering analysis

**Figure 5:**
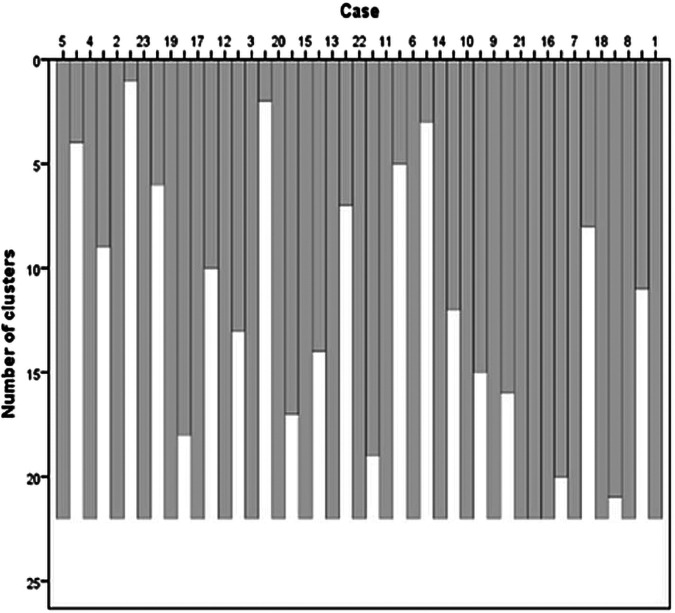
Dendrogram illustrating the results of hierarchical clustering using Ward’s method and squared Euclidean distance, identifying three distinct clusters based on energy flow variables at the ankle, knee, and hip joints during pre- and post-intervention conditions.

Hierarchical clustering using Ward’s linkage method and squared Euclidean distance grouped the PCA-derived energy flow variables into three distinct clusters, as illustrated in Figure 5.

**Cluster 1:** Hip and ankle energy inflow and outflow (pre- and post-intervention).

**Cluster 2:** Knee energy absorption (pre- and post-intervention).

**Cluster 3:** Energy generation variables observed prior to the intervention.

## 4 Discussion

This investigation examined the impacts of a six-month integrated balance and resistance training intervention on biomechanical energy transmission and practical outcomes, evaluated using the Western Ontario and McMaster Universities Osteoarthritis Index (WOMAC) among individuals diagnosed with knee osteoarthritis. The findings demonstrated significant enhancements in the biomechanical energy flow across the hip, knee, and ankle joints, concomitant with reductions in perceived pain and joint stiffness and improvements in functional capabilities as measured by WOMAC assessment scores (19, 23).

These findings highlight the strong link between improved biomechanical efficiency and positive clinical outcomes. The observed enhancements in energy transfer suggest improved joint stability, reduced mechanical loading, and increased movement efficiency during gait. The significant WOMAC score improvements further support the combined balance and strength exercise program’s therapeutic potential in alleviating OA symptoms and restoring functional independence. By demonstrating the effectiveness of this program in improving both biomechanical and patient-reported outcomes, this study underscores the value of targeted exercise interventions for optimizing joint function, reducing symptoms, and enhancing mobility in knee OA patients. These results advocate for integrating exercise-based programs into OA management and call for further research into long-term effects and personalized adaptations.

### 4.1 WOMAC score improvements

This study found a marked enhancement in WOMAC outcomes, highlighting the efficacy of the six-month regimen combining balance and strength exercises in mitigating pain and stiffness while augmenting physical function for individuals diagnosed with knee osteoarthritis. The mean WOMAC score decreased from 75.00 (pre-intervention) to 48.52 (post-intervention), with a mean difference of 26.48 (*p* = 0.009), indicating substantial clinical improvements. These improvements are crucial for enabling individuals with knee osteoarthritis to maintain an active lifestyle and enhance their overall quality of life (19).

### 4.2 Reduction in pain and stiffness

Reductions in WOMAC pain and stiffness scores are likely due to biomechanical improvements from the combined balance and strength exercise program. The program’s progressive strength, balance, and mobility exercises enhanced joint stability and reduced abnormal knee loading. By strengthening muscles and improving neuromuscular coordination, the program helped redistribute mechanical loads, alleviating stress on articular structures (7, 26, 27). Post-intervention energy flow analysis showed decreased energy dissipation at the knee, indicating minimized inefficient movement patterns. This aligns with prior research demonstrating that targeted exercise interventions improve joint mechanics and muscular support, reducing OA-related discomfort. Psychological factors, such as reduced fear of movement, may also reduce the reported pain (28).

### 4.3 Improvement in physical function

The significant improvements in the physical function domain of WOMAC further support the effectiveness of the combined balance and strength exercise program in enhancing functional mobility. Participants demonstrated increased ease in performing daily activities, such as walking and stair climbing, likely due to lower limb strength, balance, and coordination gains. Additionally, improved joint energy transfer suggests enhanced movement efficiency and reduced functional limitations.

### 4.4 Biomechanical insights from principal component analysis

The PCA revealed distinct patterns in lower limb energy dynamics, highlighting specific roles of the hip, knee, and ankle joints in energy transfer and absorption, and indicating significant adaptations following the intervention.

#### 4.4.1 Key component interpretations

i. **Principal component 1: hip-ankle synergy in energy transfer**
  PC1 captured energy inflow and outflow at the hip and ankle. This suggests a synergistic relationship between these joints in optimizing energy transfer during gait. The hip contributes to propulsion, while the ankle aids in energy dissipation. In individuals with KOA, compensatory mechanisms such as reduced knee flexion and increased reliance on the hip and ankle are common. Improved hip-ankle coordination may help mitigate these compensations by enhancing propulsion and stability. The exercise program’s emphasis on strengthening and stabilizing these joints likely plays a key role in this improvement, reducing stress on the knee and improving overall gait efficiency. These values also indicate stable, well-distributed energy dynamics before the intervention.
ii. **Principal component 2: knee’s role in shock absorption**
  PC2 correlated strongly with knee energy absorption, highlighting the knee’s primary role as a shock absorber during gait. The observed post-intervention decrease in energy absorption suggests reduced knee stress, likely contributing to significant WOMAC pain score improvements. The combined balance and strength exercise program achieves this by strengthening the quadriceps and hamstrings, improving joint stability, and promoting more efficient movement patterns. These changes reduce the knee’s burden during weight-bearing activities, aligning with the program’s goal of reducing joint loading and improving functional outcomes in KOA patients. The knee serves as a key shock-absorbing structure responsible for managing the dissipation of energy throughout the weight acceptance and stance phases of the gait cycle. These findings align with the understanding that the knee is crucial in mitigating impact forces and preserving stability during movement.
iii. **Principal component 3: differential energy generation across joints**
  PC3 reflected variations in energy generation across the hip, knee, and ankle, underscoring the differential contributions of these joints to energy production during gait. In KOA, the knee often exhibits reduced functional capacity, including diminished strength and range of motion. As a result, the hip and ankle play a more significant role in energy generation. The combined balance and strength exercise program’s focus on strengthening the hip and ankle compensates for the knee’s limitations, thereby supporting improved gait mechanics. PC3 also reflects secondary energy redistribution patterns, possibly involving energy transfer between joints or compensatory mechanisms during gait. These patterns could represent energy redistribution from the knee to other joints (e.g., ankle or hip) to maintain balance or adapt to changes in gait mechanics. For instance, an increased knee absorption post-test might indicate a shift in energy management strategies to reduce joint loading or improve efficiency.
iv. **Principal component 4: localized dynamics and individual variations**
  PC4 captured localized energy dynamics, focusing on knee absorption and other joint-specific variables. This suggests that PC4 represents subtle, joint-specific adjustments in energy flow. These patterns may reflect individual variations in joint stability, muscle strength, or movement strategies observed post-intervention. While these variations could be influenced by disease severity, adherence to the combined balance and strength exercise program, or pre-existing biomechanical differences, further research is needed to explore the sources of these variations and their implications for personalized rehabilitation strategies in KOA management. These localized dynamics may reflect fine-tuning energy flow at specific joints to optimize movement efficiency or adapt to external demands. For example, reduced knee absorption in PC4 could indicate a shift toward more efficient energy utilization at the hip or ankle.

#### 4.4.2 Overall trends from rotated component matrix

Analysis of the rotated component matrix highlighted several overall trends in energy flow following the intervention. Post-test conditions showed a general increase in energy inflow and outflow at the ankle, knee, and hip joints (e.g., ankle inflow post-test: 0.851; hip inflow post-test: 0.841), suggesting enhanced energy transfer efficiency following the combined balance and strength exercise program intervention. While pre-test energy generation was prominent at the ankle and knee (e.g., ankle generation pre-test: 0.897; knee outflow pre-test: 0.897), post-test data indicated a shift in energy generation patterns, particularly at the hip (hip generation post-test: −0.601), possibly due to improved joint coordination and reduced reliance on specific joints for energy production. Furthermore, post-test conditions revealed decreased energy absorption at the knee (knee absorption pre-test: −0.898; post-test: −0.741), indicating reduced energy dissipation and potentially improved energy conservation during gait.

### 4.5 Hierarchical clustering and integrated biomechanical patterns

Hierarchical clustering complemented the PCA findings by grouping energy flow variables into three distinct clusters, revealing meaningful lower limb energy dynamics patterns that align closely with biomechanical function and joint-specific roles during gait.

#### 4.5.1 Cluster interpretations

i. **Cluster 1: hip and ankle energy inflow/outflow**
  This cluster, encompassing hip and ankle energy inflow and outflow (pre- and post-intervention), highlights the coordinated function of the hip and ankle joints in mechanical energy transfer. These joints collectively contribute to propulsion and postural stability, working synergistically during gait. The hip acts as a major generator of forward momentum, while the ankle is critical for terminal stance push-off. This synergy aligns with prior findings identifying the hip and ankle as key contributors to energy generation and efficient movement in healthy and pathological gait patterns.
ii. **Cluster 2: knee energy absorption**
  This cluster, defined by knee energy absorption (pre- and post-intervention), reflects the knee’s essential role in shock absorption and energy dissipation during the stance phase. It underscores the joint’s function in modulating impact forces. In individuals with knee osteoarthritis, altered energy absorption at the knee may serve as a compensatory mechanism to reduce joint loading or pain. Effective shock absorption is critical for preserving balance and joint integrity.
iii. **Cluster 3: pre-intervention energy generation**
  This cluster, comprising energy generation variables observed prior to the intervention, may reflect compensatory strategies developed in response to KOA-related impairments. The dominance of pre-test variables suggests a baseline pattern of altered energy generation before rehabilitation. Individuals with KOA may rely more heavily on the hip or ankle to compensate for reduced function at the knee. Such adaptations, including proximal energy generation, are frequently reported in pathological gait and serve to reduce joint stress or improve efficiency.

#### 4.5.2 Alignment with PCA and adaptive changes

The clustering results exhibit strong correspondence with the PCA-derived components, providing an integrated representation of how different joints contribute to lower limb energy management and how these contributions evolve in response to rehabilitation. Cluster 1 parallels Component 1 (C1), which captured coordinated hip and ankle inflow/outflow patterns, emphasizing their central role in energy transfer. Cluster 2 aligns with Component 2 (C2), reflecting the knee’s dominant role in energy absorption. Cluster 3 shares features with Components 3 and 4 (C3 and C4), which describe pre-intervention energy generation and post-intervention redistribution patterns. Together, these analyses quantify variance and visualize relationships, revealing joint-specific energy flow patterns and their adaptation during the intervention.

The adaptive movement dynamics suggested by Cluster 3, and generally observed across the PCA components, likely reflect compensatory neuromuscular strategies to address joint dysfunction or pain, particularly in knee osteoarthritis patients. These adaptations appear to involve three key biomechanical adjustments: increased hip energy generation to compensate for diminished knee function, thereby maintaining forward momentum while reducing knee joint loading; modified ankle push-off mechanics characterized by greater plantarflexor contribution to facilitate propulsion and further offload the affected knee during terminal stance; and systematic redistribution of energy absorption and generation patterns across the lower limb kinetic chain, effectively dispersing mechanical stress away from the pathological knee—a well-recognized protective mechanism in KOA gait. Together, these compensatory strategies demonstrate the neuromuscular system’s remarkable capacity to reorganize movement patterns in response to joint pathology while preserving overall locomotor function.

### 4.6 Relationship between WOMAC and energy flow dynamics

The relationship between WOMAC scores and biomechanical energy flow provides crucial insight into how joint mechanics influence functional performance. The in-depth analysis of energy flow dynamics derived from PCA and clustering further illuminates these connections.

**Pain reduction and energy efficiency:** A decrease in WOMAC pain scores suggests improved joint function and reduced compensatory movement patterns. Lower pain levels correlate with decreased energy dissipation at affected joints, particularly the knee, allowing for more efficient movement. Reduced negative joint power indicates less energy absorption due to excessive braking forces (5, 29). Improvements in muscle activation patterns once the pain is reduced further enhance efficiency (30).

**Stiffness reduction and improved joint coordination:** Lower WOMAC stiffness scores suggest increased joint mobility, which enhances energy transfer across segments. A decrease in stiffness allows for smoother intersegmental energy flow, reducing abrupt deceleration forces and contributing to joint stability (31–33). This is reflected in greater positive joint power at the hip and ankle, enabling more efficient propulsion, a finding corroborated by PC1’s focus on hip-ankle synergy and Cluster 1.

**Functional improvement and enhanced energy transfer:** Improvements in the WOMAC function domain indicate better motor control and coordination. Enhanced energy redistribution between joints, as seen in PC3, PC4, and Cluster 3, reflects more effective gait mechanics and reduced compensatory strategies. Increased symmetry in energy flow between limbs signifies balanced load distribution and improved functional capacity (5, 7).

This connection underscores the reliability of energy flow analysis in evaluating movement efficiency and functional improvements, indicating that a reduction in pain may correlate with increased energy generation at the hip. In contrast, improved function may correlate with more efficient energy transfer between the knee and ankle. These altered patterns may reflect compensatory mechanisms adopted by individuals with knee OA, which are effectively captured by the PCA and clustering results.

### 4.7 Validity of energy flow analysis using WOMAC

The strong correlation between WOMAC improvements and changes in energy flow supports the validity of biomechanical energy flow analysis as an objective measure of functional recovery. By demonstrating how WOMAC outcomes align with the detailed biomechanical findings from PCA and hierarchical clustering, this study strengthens the credibility of energy flow analysis as a reliable marker for assessing movement mechanics and rehabilitation efficacy (34). The insights gained from the dominant roles of hip and ankle energy inflow/outflow, the knee’s critical function in energy absorption, and the complementary nature of pre- and post-intervention energy flow patterns underscore the utility of these analytical methods.

### 4.8 Clinical implications

The biomechanical insights have direct clinical implications for KOA management. Increased energy transfer efficiency, especially the hip-ankle synergy, and reduced knee absorption post-intervention indicate improved gait mechanics and reduced joint loading. This directly translates to enhanced mobility and reduced pain in KOA patients. The correlation between WOMAC improvements and energy flow supports using energy analysis as an objective recovery metric for clinical assessment.

As revealed by PCA and clustering, the distinct roles of joints in energy management highlight the need for joint-specific interventions. For instance, strengthening the hip and ankle could significantly enhance energy generation and propulsion, while strategies to reduce knee loading (e.g., through improved quadriceps and hamstring strength) may improve absorption efficiency and pain. Understanding compensatory mechanisms, such as increased hip energy generation or modified ankle push-off mechanics, helps clinicians tailor rehabilitation to address specific joint deficits and leverage existing adaptive strategies (5).

### 4.9 Strengths and limitations

The study employed robust techniques, such as PCA and hierarchical clustering, to identify complex energy flow trends and provide a comprehensive understanding of lower limb biomechanics. These methods offered complementary insights, quantifying variance and visualizing relationships effectively. However, limitations include focusing solely on three major joints and a relatively small sample size. Future studies should include larger cohorts, consider additional anatomical regions beyond the hip, knee, and ankle, and investigate a wider range of activities.

### 4.10 Future directions

Future research should expand sample sizes and integrate multimodal biomechanical assessments to build upon these findings. Key areas include examining age-related variations in energy flow, optimizing training regimens based on specific energy patterns, analyzing sport-specific movements, and leveraging machine learning approaches to identify subtle biomechanical signatures of recovery. Furthermore, energy flow analysis has potential in broader applications beyond OA, offering valuable insights for rehabilitation in various neuromuscular disorders, injuries, and age-related mobility decline.

## 5 Conclusions

This study demonstrated that a six-month combined balance and strength exercise program induced significant alterations in mechanical energy flow across the hip, knee, and ankle joints in individuals with knee osteoarthritis, reflecting adaptations in energy dynamics throughout the gait cycle. Principal Component Analysis (PCA) effectively reduced data dimensionality and uncovered latent structures within energy flow variables, offering a nuanced understanding of joint-specific biomechanical responses to the intervention.

These findings underscore the utility of PCA in biomechanical research and reinforce the role of targeted exercise programs in enhancing joint coordination and energy efficiency. The results contribute to the growing body of evidence supporting exercise-based rehabilitation for knee OA and suggest that energy flow metrics may serve as objective indicators of functional improvement.

Future research should examine the long-term sustainability of these biomechanical adaptations, explore the relationship between energy flow and clinical outcomes in more diverse populations, and consider integrating plantar pressure measurements with energy flow analysis to evaluate kinetic chain efficiency comprehensively.

## Data Availability

The raw data supporting the conclusions of this article will be made available by the authors, without undue reservation.
